# Get up, stand up: a randomized controlled trial to assess the effectiveness of a messenger-based intervention to reduce sedentary behavior in university students

**DOI:** 10.1007/s10389-022-01747-7

**Published:** 2022-08-16

**Authors:** Mona Kellner, Franziska Faas

**Affiliations:** Department of Sports and Sports Science, Heidelberg University, Heidelberg, Germany

**Keywords:** University health promotion, sedentary behavior, university students, messenger-based intervention, prolonged sitting, light intensity physical activity

## Abstract

**Aim:**

Sedentary behavior is a severe and independent risk factor for health. According to current research, sitting time is at a dangerously high level. Especially young adults show a high prevalence compared to others. The study aimed to assess the effectiveness of a 6-week messenger-based intervention to reduce sedentary behavior in university students.

**Subject and methods:**

The 345 university students that enrolled were randomly assigned to the intervention group (*n* = 173) and control group (*n* = 172). Randomization and allocation to the trial group were computer assisted. The trial was conducted remotely, without any personal contact. A drop out of 276 participants led to a primary analysis of 71 (IG *n* = 41; CG *n* = 31) participants. Sedentary behavior was assessed online using the Heidelberg Questionnaire for the Assessment of Sitting Behavior, at 5 time points: baseline (T0), 2 weeks (Z1), 4 weeks (Z2) 6 weeks (end of the intervention, T1), and follow-up 4 weeks after intervention (T2).

**Results:**

Mixed ANOVA was carried out for T0 and T1 to reveal interaction effects between time and group. Mean differences show a highly practically and statistically relevant reduction in sitting time in the intervention group of 60 min between baseline and T1. No sustained effect of the intervention could be detected by analyzing sitting times at follow-up, 4 weeks after the end of the intervention.

**Conclusions:**

Reduction in sedentary behavior in the intervention group after 6 weeks shows that the intervention is practically and statistically relevant. Limitations concerning the assessment method (questionnaire) as well as the sample size should be considered. The trial serves as a pilot study. However, the positive outcome of sitting time reduction paves the way for further research in this field.

**Supplementary Information:**

The online version contains supplementary material available at 10.1007/s10389-022-01747-7.

## Introduction

The effects of physical activity on human health have been an important field of research in public health for several decades. In the past few years, sedentary behavior has become a focus of research interest in this field. The scientific activities of the past years refer to the research of the direct effects of sedentary behavior on the development of non-communicable diseases (NCD) as well as the occurrence of premature death.

The Sedentary Behavior Research Network (SBRN) defines sedentary behavior as activities that are performed in a seated or reclined position and do not exceed a maximum energy expenditure of 1.5 MET (Metabolic Equivalent of Task, a unit to report the expansion of energy during physical activity) (Tremblay et al. 2017). This low energy expenditure appears to be a major cause of the degenerative effects of sedentary behavior (Manini et al. 2006). The lack of utilization of body systems during sedentary time arguably leads to metabolic, hormonal, and muscular imbalances, which may attenuate the anti-inflammatory effects triggered by human musculature and promote systemic dysfunction (Pedersen 2007). As a result of increased sedentary behavior, muscular problems, as well as non-communicable diseases (NCDs), such as cardiovascular diseases, diabetes, osteoporosis, and even cancers can occur (Biswas et al. 2015). An analysis of data gathered by the US National Health and Nutrition Examination Survey (NHANES) shows that the adult population spends an average of 42% of a 24-h day seated. Isotemporal substitution of sedentary behavior, however, could reduce the likelihood of premature death (Clarke and Janssen 2021). In contrast, sedentary behavior appears to be an independent risk factor for the health impairments mentioned earlier. Thus, if daily sedentary time is not reduced, but only more physical activity (MVPA) is performed, the health risk from sitting hardly changes (Koster et al. 2012).

Globally, 3.8% of all-cause mortality can be attributed to increased sedentary time (Rezende et al. 2020). The consensus in current research therefore is that increased sedentary behavior carries a high risk for premature mortality as well as NCD and is correlated with all-cause mortality (Chau et al. 2013; Schmid et al. 2015; Rezende et al. 2020; Clarke and Janssen 2021). However, there is disagreement on the cut-off value of daily sitting time, i.e., the value above which daily sitting becomes a health risk. In a recent review detrimental effects were detected during a daily sitting time of more than 3 hours (Rezende et al. 2020). In other papers it became apparent that the cut-off value is determined at about 8 hours per day (h/d) (Schmid et al. 2015) or a 5% higher risk of all-cause mortality for each additional hour of sitting above a 7-hour daily sitting time (Chau et al. 2013).

A recent global review reported a median of 4.7 h/d of average total sitting time for adults across all included countries (Mclaughlin et al. 2020). This result is consistent with the findings of the scoping review by Rezende et al. (2020), who determined a median average sitting time of 5 h/d in data from 54 countries worldwide. National surveys such as the US National Cohort Study indicate an average of 752.4 minutes per day (min/d; 12.54 h/d) and therefore are in a marked contrast to these findings. The latest health report of a German health insurance company also shows worrying sitting times of an average of 523 min/d (8.71 h/d). Young adults between the ages of 18 and 29 are particularly affected. People in this age group show the highest sitting times. According to this 74% of the age group sit 8 h or more per day. The average daily sitting time here is 605 min/d (10 h/d), out of which 2 h per day are spent sitting in front of electronic media (Froböse and Wallmann-Sperlich 2021).

A survey of children’s and adolescents’ sitting times from 2017 shows that sitting time also increases significantly with rising grade level and that a large part of daily sitting time can be attributed to school activities (Huber and Köppel 2017). Congruent to everyday school life, everyday student life is also characterized by sedentary activities: university events such as lectures and seminars, as well as writing term papers, learning and preparing presentations take place while sitting and nowadays primarily in front of a computer.

Furthermore, it can also be assumed that young people’s leisure time is also primarily spent sitting related to the almost invariably digital lifestyle. A nationwide survey of students in Germany from 2018 revealed that almost half of the respondents (42%) spend between 8 and 12 h a day seated during lecture time. Respondents also reported they often sit for long periods at a time, which may well be another risk factor for health (Spin Sport Innovation and Constata 2018). Measures to curb the Covid-19 pandemic also appear to have contributed to further increases in sitting time, particularly in this age group, through even more remote working and physical distancing (Bates et al. 2020; Zieff et al. 2021). It can be assumed that the activities of daily living (ADL) have decreased significantly due to working and studying from home. This means that physical activities that do not represent a sporting activity but are carried out in order to cope with everyday life, are significantly lower or are no longer carried out at all, such as walking to the university/ to the library/ to friends. The energy expended in these everyday activities is also called NEAT (non-exercise activity thermogenesis). NEAT, in addition to the resting metabolic rate, accounts for a significant portion of a person’s total daily energy expenditure. If the accomplishment of some ADL is eliminated because the majority of the day can be spent sitting, adverse metabolic, cardiovascular, and muscular profiles can result (Levine and McCrady-Spitzer 2018).

Recent research clearly shows that young adults spend the most time sitting in comparison to other groups and therefore represent an extremely vulnerable group with a high-risk profile. The empirical figures presented illustrate the high relevance of interventions to reduce sedentary behavior among students. The high rate of electronic media use among young adults is a factor which needs to be considered concerning the strategy to address and approach the target group (Feierabend et al. 2019). Health promotion interventions in particular must be designed attractively in order to reach many people. In times of a pandemic, digital interventions are of great advantage because face-to-face supervision is not necessary, making it easy to comply with the stipulation of contact restrictions. The use of smartphones, messenger services, and social media is detected at almost 100% within the group of young adults, and therefore makes a digital intervention to reduce sitting time essential (Feierabend et al. 2019; Statista 2020a, b). As the smartphone has seemingly become an everyday companion of young individuals, there is high potential to use digital interventions to effectively change health behaviors, specifically sedentary behaviors, that occur throughout daily life.

For the reasons stated above, a digital intervention was designed to reduce sedentary behavior in students. The aim of the survey was to test the effectiveness of a digital intervention to modify sedentary behavior in the target group of students. During the time of the intervention (November 2020), the University of Heidelberg was closed, and all participating students had to work remotely. Thus, the need for the intervention was considered as high. The initially high interest to participate in the study could prove this assessment. Due to this situation, the effectiveness of the intervention was assessed as high.

## Methods

### Recruitment

Following a positive ethics vote by the responsible ethics committee at Heidelberg University, the study was advertised at Heidelberg University. Information about the study was disseminated via social media channels, websites, and e-mail distribution lists of individual institutes at Heidelberg University. To participate in the study, individuals had to be students enrolled at Heidelberg University and in possession of a smartphone in order to receive the short messages included in the intervention.

Interested students had the opportunity to register for the study on an online portal and conducted the baseline measurement in the form of an online survey right after the registration. Participants gave informed consent online by downloading the informed consent form and by clicking the “consent button” before completing the registration and the baseline measurement. The consent was designed as a mandatory field so that the registration could only occur by agreeing to the informed consent form. Accordingly, this is an ad-hoc sample of students from all disciplines at Heidelberg University.

### Procedure

After the enrollment period, computer-assisted randomization of all enrolled participants into intervention and control groups was performed. During the following 6 weeks the intervention group received messages in the style of action and coping plans that encouraged sitting time interruption. The control group only received healthy lifestyle information without any behavior change prompts. After randomization, participants were added to the respective message channel.

## Data collection and measures

### Primary outcome measure

The primary outcome measure was the sedentary behavior of the sample, which was assessed using the Heidelberg Questionnaire for the Assessment of Sitting Behavior (Lerchen et al. 2016).

### Secondary outcome measure

Physical activity time was also assessed by the Heidelberg Questionnaire for the Assessment of Sitting Behavior. Because physical activity is not directly addressed through the intervention, it is considered as a secondary outcome variable. Furthermore, the questionnaire was extended by 10 items (Schwarzer et al. 2007) to assess action and coping planning. The added items provide information about whether the respondents created action plans during the intervention and whether they were able to overcome barriers in order to carry out the activity anyway (coping plans).

The questionnaire was completed online by the respondents. At the respective measurement time points, the participants were informed by short message that they had to complete the questionnaire and were directed to the survey via a link. After the baseline measurement (T0) before the start of the intervention, two intermediate measurements, 2 (Z1) and 4 weeks (Z2) after the start of the intervention, were conducted. The final measurement (T1) took place 6 weeks after the beginning of the intervention. A follow-up measurement (T2) was also conducted 4 weeks after the end of the intervention to verify a sustained effect of the intervention. As the effect of the 6-week intervention is of interest here, analysis for the outcome variables is analyzed between measurement time points T0 (baseline) and T1 as well as between T1 and T2 for the detection of sustained effects.

### Data/statistical analysis

**Fig. 1:**
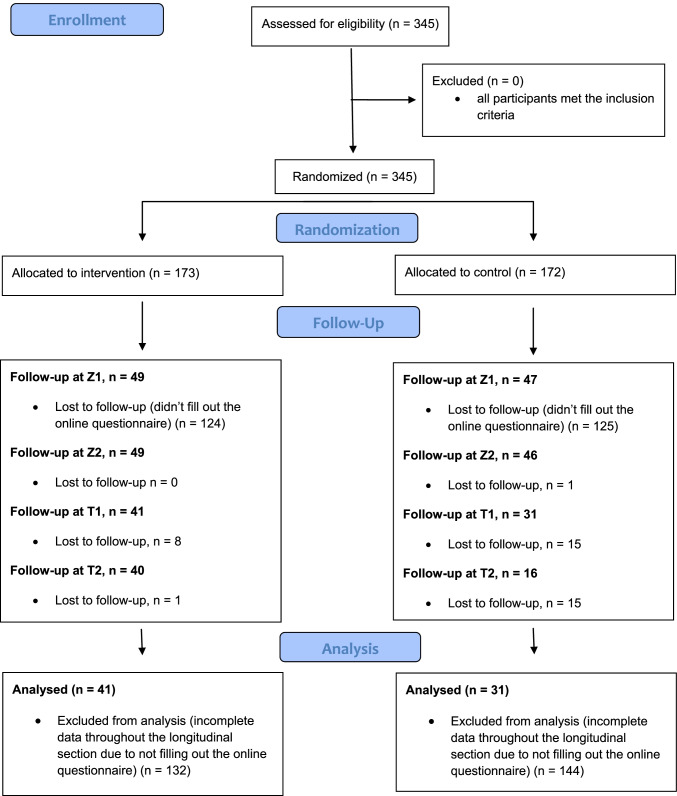
CONSORT flow diagram

The initial data set included 345 participants who were part of the ad hoc sample after the baseline measurement (T0). The participants were randomly assigned to the intervention group and control group (IG_T0_ *n* = 173; CG_T0_ *n* = 172). Due to drop outs after the start of the intervention, only 103 individuals participated in the online survey at Z1, 2 weeks after the start of the intervention. During the course of the intervention, another 31 persons dropped out, resulting in a final data set of *n* = 72 participants (IG *n* = 41; CG *n* = 31), which was integrated into the data analysis using IBM SPSS Statistics 26 (see Fig. 1). For text message-based interventions, no data concerning dropout rates is available to date. A recent review on dropout rates in app-based interventions for chronic disease shows an average of 43% of dropout (Meyerowitz-Katz et al. 2020).

The increased dropout rate in the case of the present study can probably be attributed to the digital nature of the intervention. Recruitment as well as enrollment and measurement were carried out anonymously and online so that no personal contact was held with any of the participants. The physical distance throughout the study was necessary due to the lockdown situation because of the Covid-19 pandemic. In addition to the positive aspects of digital interventions already described, the methodology may nevertheless result in a certain anonymity, which could promote drop out from the intervention. In the follow-up study (T2), four weeks after the intervention, a response rate of 76.39% was achieved.

The questionnaire was used to survey sitting and physical activity times in the university context and within different everyday domains. Sitting and movement times were surveyed within the given domains (when eating, working at home and at the university, for transport and during leisure time) on a typical day during the week and on a typical weekend day. For simplified analysis of the collected data, a weighted total sedentary time as well as total physical activity time were calculated for each of the measurement time points. For all variables, interaction effects between the groups and over time were analyzed using single-factor analysis of variance in a mixed-design (mixed ANOVA). As preconditions for mixed ANOVA could not be reached in the data for sitting at work, a change score for this variable was calculated. A one-sided, independent sample t-test of the change score was computed to detect effects between the groups and over time. The level of significance was set at *p* < .05.

### Intervention

The intervention consisted of instant-messages that prompted participants to take breaks from sitting and engage in low-intensity physical activity. The messages were designed in the style of if-then plans, and thus aimed to increase participants’ action and coping planning.

Participants in the intervention group received two messages per day (in the morning and in the evening) that included direct prompts to interrupt sitting with specific examples of isotemporal substitution of sitting. The participants were thus presented with action plans for the time during work or their studies, as well as implementation strategies for everyday activities during leisure time.

The control group, however, only received messages with content informing them about a fundamentally healthy lifestyle.

## Results

### Sample demographics

After inclusion of all relevant survey questionnaires, the sample can be characterized as follows: The population consisted of 88.9% female and 11.1% male study participants. In terms of age structure, 44% of the participants were between 21–24 years old, and this age group was the largest component of the evaluation. According to the Social Survey of the German Student Union in 2016, the average age of German students is 24.7 years old, which means that the present sample is comparatively young (Middendorff et al. 2017). This may be due to the fact that predominantly first-year students participated in the survey; 52.8% of the students stated that they were currently in their 1st–3rd semester, 27.8% of the participants were in their 4th–6th semester. Only 18.1% of all participants were studying beyond the 6th semester. Data analysis of disciplines shows participation of students from all disciplines with a focus of participating students from Behavioral and Empirical Cultural Studies (52.8%). Randomization into intervention and control groups occurred after participant registration and concurrent baseline survey. In the final data set, the intervention group consists of *n* = 41 participants, the control group of *n* = 31 participants.

### Baseline differences between groups

The two-sample t-test shows that randomization was successful with only one significant difference between groups: the control group showed higher sitting times in the domain *working at home.*

### Primary outcome

The baseline data of the total sample show that the participating students spent an average of 11.57 h/d (SD ± 2.21) sitting at the beginning of the intervention (T0). At the beginning of the survey, the average time spent sitting to do their studies was 5.65 h/d (SD ± 1.75). This may imply that the proportion of working time spent sitting accounts for almost half of the total daily sitting time (48.83%).

Table 1 Differences in the collected sitting times between the measurement time points in intervention and control group in minutes.

**Table 1:** Assessed sitting times in intervention and control group at T0, T1, and follow-up and the mean difference between the time points

	Baseline T0 [h:mm ± SD]	T1: 6 weeks [h:mm ± SD]	Follow-up: 4 weeks post intervention [h:mm ± SD]	d_1_ * [min]	d_2 *_ [min]
Intervention group	11.95 ± 2.20	10.95 ± 2.12	10.98 ± 2.58	- 60	+ 1.8
Control group	11.07 ± 2.16	11.31 ± 2.14	11.12 ± 2.27	+ 14.4	- 11.4

*d_1_ and d_2_ are calculated differences between the mean values at each time points

The main interest of the study lies in the identification of effects concerning the sitting time due to the 6-week intervention. Therefore, a mixed ANOVA for measurement time points T0 and T1 was carried out. The analysis reveals a significant interaction effect between time and group assignment F (1, 70) = 4.25; *p* = .043, partial η^2^ = .06. Total sitting time in the intervention group decreases by 60 min from pre- to post measurement (T0 M = 11.95 (SD ± 2.20); T1 M = 10.95 (SD ± 2.12)), representing both a statistically significant and highly practically relevant result. The sitting times in the control group increase by 14.4 min within the 6-week intervention (T0 M = 11.07 (SD ± 2.16); T1 M = 11.31 (SD ± 2.12)).

**Fig. 2:**
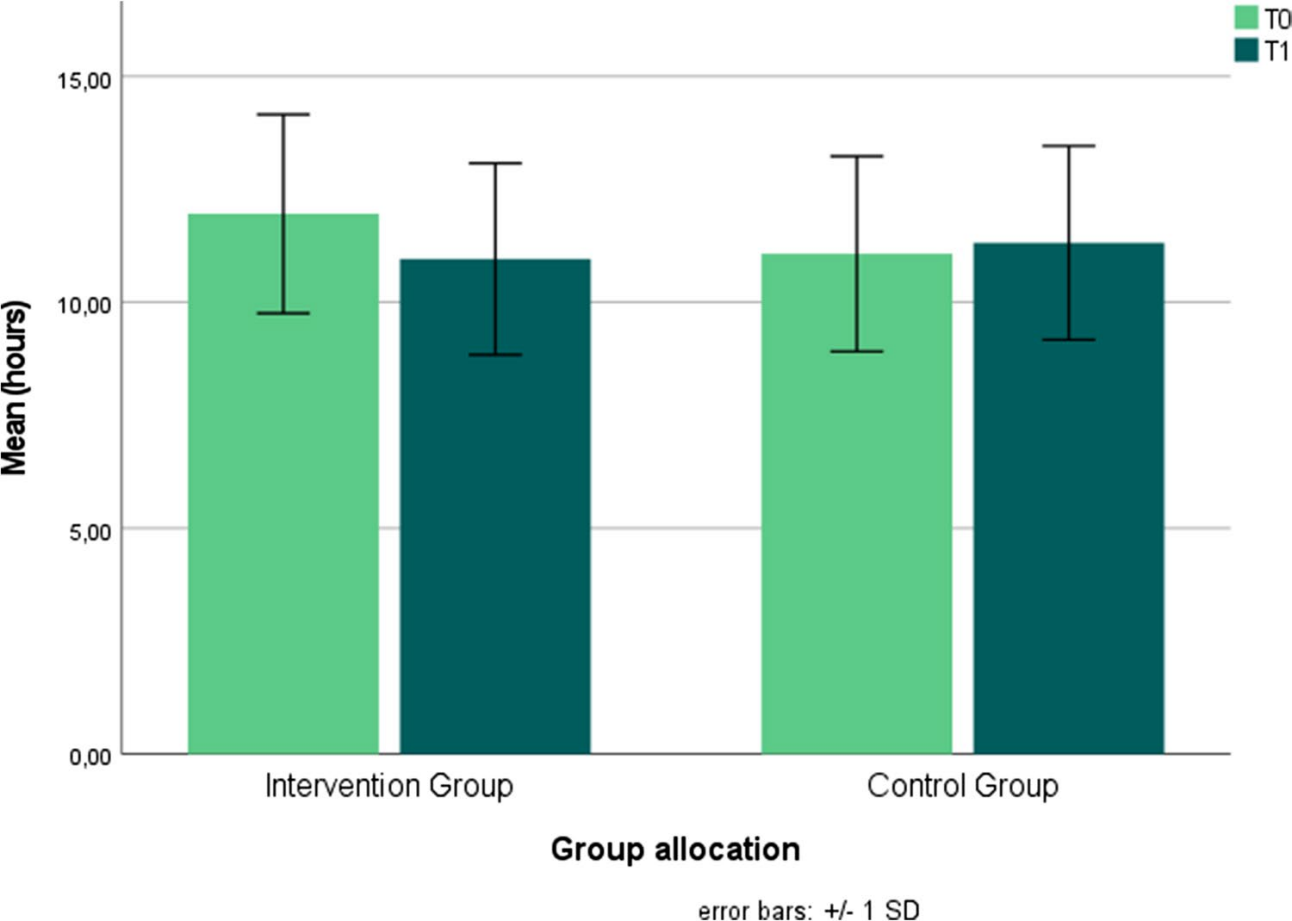
Representation of sitting times in intervention and control groups at baseline and final measurement time points

In the domain working, a reduction of sitting time is shown in the intervention group with 44.4 min (T0 M = 5.99 (SD ± 1.77); T1 M = 5.25 (SD ± 1.91)), whereas an increase of 28.2 min sitting time is to be found in the control group in this domain (T0 M = 5.21 (SD ± 1.66); T1 M = 5.68 (SD ± 1.18)). To analyze the change of sedentary behavior during working, the change score in sitting time between T1 and T0 was calculated. An independent sample t-test (one-sided testing) reveals a significant change of sedentary behavior between IG and CG over time with a small to moderate effect size: one-sided *p* = .007; 95% CI [–1.069 to –0.115], Cohen’s d = –.594. The changes in total sitting time between T0 and T1 can be seen in Fig. 2.

When looking at the follow-up to test for possible sustainability of change in sitting due to the intervention, the following can be stated: Within sitting times, an increase of 1.8 min can be seen in the intervention group compared to measurement time T2; a reduction of 11.4 min in the control group.

For the detection of interaction effects between T1 and follow-up, mixed ANOVA was carried out. The differences are not statistically significant: F (1,52) = .585, *p* = .448, partial η^2^ = .011

### Secondary outcome

Regarding the total sample, physical activity times of 2.77 h (SD ± 1.34) per day on average can be recorded at T0. Within the framework of the comparative tests of the physical activity times between the intervention and control groups, it may be stated that no interaction effects between intervention and control group and over time could be detected: F (1,70) = .005, *p* = .942, partial η^2^ = .000. Mixed ANOVA between T0 and T1 was computed for this comparison.

Comparison of T1 and T2 were computed using mixed ANOVA to identify a possible change 4 weeks after the intervention. The physical activity times in the intervention group increase by 26.4 min, in the control group by 1.8 min. No significant difference could be detected in physical activity times: F (1,56) = .524, *p* = .468, partial η^2^ = .009.

The action and coping planning data, which were asked in an additional 10 items, were added to provide an overview of the intention to change behavior during the intervention period. Mixed ANOVA was carried out for both variables, action and coping planning between T0 and T1, as well as between T1 and T2 (follow-up). The analysis in the variable of action planning does not show an interaction effect throughout the intervention F (1,70) = .173, *p* = .679, partial η^2^ = .002; or between T1 and follow-up: F (1,57) = .239, *p* = .672, partial η^2^ = .004.

Only the coping planning differed significantly between group assignment and over the intervention time. Coping planning increased significantly in the IG compared to the CG with a small to moderate effect: F (1,70) = 4.015, *p* = .049, partial η^2^ = .054. The data at follow-up do not differ significantly from T1: F (1,57) = 2.435, *p* = .124, partial η^2^ = .041.

## Discussion

The baseline measurement of the study already shows that sitting time of the participating students is on an extraordinarily high level. According to the data, students have a strongly sedentary lifestyle, which is characterized by an average sitting time of 11.57 h/d (SD ± 2.21). This fact is of particular concern in times of remote working in terms of the increased risks of disease due to highly increased sitting time (Chau et al., 2013; Rezende et al., 2020; Schmid et al., 2015).

According to this, the data indicates the high relevance of digital interventions. The significant reduction in sitting time in the intervention group between T0 and T1 indicates the effectiveness of the intervention. Within the intervention group, the reported sitting time decreases by an average of 60 min between T0 and T1. Regarding the finding that all-cause mortality increases by 5% for each additional hour above a total daily sitting time of 7 h/d (Chau et al. 2013), the reduction of 60 min per day is a clear and practically relevant success. In contrast, an increase in sitting time of 14.4 min is evident in the control group. Additionally, the action planning in both groups does not differ significantly throughout the intervention. Only the coping planning increased significantly in the intervention group over the 6-week intervention. This could indicate that messages need to be adapted in terms of motivation formation to also promote action planning and therefore support intention formation and action execution.

The study can be classified as an initial pilot study, which also demonstrates the feasibility of digital sedentary time reduction in particular. The overall reduction in sitting time is mainly due to the reduction in sedentary time during university work. This shows that the intervention was well tailored and reached participants in a place where they spend the most time sitting.

The approach of digital health promotion by means of short message-based movement promotion in everyday life to reduce sedentary behavior holds high potential, especially for the target group of students. The low statistical strength of evidence can be attributed to the nature of the data collection, which includes some strengths and weaknesses.

## Limitations and strengths

The sitting behavior of the participating students in the present study was surveyed using the Heidelberg Questionnaire for the Assessment of Sitting Behavior. The advantage of this survey method is that it can be used cost-effectively for a large sample. The questionnaire has a test-retest reliability of r = 0.9 (*p* < .001), which shows high reliability. Criterion validity was tested through a comparison of the subjective data of the questionnaire and objective data, measured with ActiGraph GT3X+, which was shown to be a very valid tool for the measurement of physical activity and sedentary behavior. Results show a significant validity for the sedentary times on weekdays of r = .35 (*p* = .003), the weekend-data as well as the physical activity data only show a non- significant tendency toward a positive correlation (Lerchen et al. 2016).

However, the survey method also means that all data are subjective. It is known from previous studies that the estimation of one’s own time spent sitting is often subject to a significant underestimation (Urda et al. 2017; Aunger and Wagnild 2022). In addition, the information was provided retrospectively, in relation to the past 2 weeks. For this reason, recall bias cannot be ruled out entirely.

The items of the questionnaire are structured in such a way that the sitting times in the respective domains could be specified in 0.5-hour increments. Potential sitting breaks of less than 30 minutes were therefore not recorded. In addition, only the sitting times per se were queried, but not specifically the sitting breaks. Thus, no statement can be made as to whether the participants implemented the sitting breaks in the course of the six-week intervention. However, the reduction in sitting time in the intervention group between the baseline and the final measurement, which averaged 60 minutes, indicates that the interruptions in sitting time must have increased.

Furthermore, we did not ask the participants if they use smartwatches or any similar digital device to increase physical activity in their everyday life. Consequently, the influence of a potential additional reminder to move more and sit less through a smartwatch can not be ruled out.

The strengths of the study are to be found in the proven feasibility of the intervention as well as the tendency to change behaviors through the digital intervention. In times of advancing digitalization and technologization, and in special situations such as that of the Covid-19 pandemic, special measures must be taken to reach the target group with health promotion offers.

Digital, low threshold offers are enormously important for health promotion in the target group of students, which consists primarily of young adults. Evidence already presented in the field of e-health shows that good results can be achieved through digital health measures (Kellner 2021). This evidence should also be advanced in the promotion of everyday activity. The present results can provide direction for this.

## Conclusion and future directions

The study to assess the effectiveness of a messenger-based intervention to reduce daily sedentary behavior in students is, to our knowledge, one of the first studies on this topic. A study from Canada also piloted the effectiveness of a messenger-based intervention on the frequency and length of students’ sedentary breaks and low-intensity and moderate physical activity (Cotten and Prapavessis 2016) and has already demonstrated initial positive effects. There is broad evidence on the topic of digital health promotion, but messenger technology has not yet been used to promote basic, broad-based health promotion in students’ daily lives (Kellner 2021).

The present study may be regarded as a first pilot study, which is intended to highlight the importance of the topic. Initial results of the study can be considered groundbreaking. The findings of the survey can indicate to what extent the effort for further studies in this field should be made.

A clear tendency in the direction of a reduction of sedentary behavior of the intervention group is recognizable. Following the state of research in the field of digital health promotion (e-health), further studies should be conducted to prove the effectiveness of the interventions in the field of sedentary behavior reduction and promotion of light-intensive physical activity.

The various advantages of addressing the young target group digitally are obvious. One positive aspect is the high user rate of digital devices and messenger services among people in our target group. It should be mentioned here that positive effects of digital health promotion measures in the e-health sector have already been researched.

Since the limitations of our study do not allow us to make a general statement, we must now examine in further steps the extent to which the addressees also implement the prompts conveyed in the short messages and sustainably integrate them into their everyday lives. Ideally, the intervention should be tested using objective measurement methods, such as actigraphy, so that the occurrence of confirmation bias and recall bias can be prevented.

## Supplementary information

ESM 1 (DOC 95.5 kb)

ESM 2 (DOC 96 kb)

## Data Availability

Not applicable.
