# Supplementary material for: Get up, stand up: a randomized controlled trial to assess the effectiveness of a messenger-based intervention to reduce sedentary behavior in university students
Source: Z Gesundh Wiss. 2022 Aug 16:1–9. Online ahead of print. doi: 10.1007/s10389-022-01747-7 (PMC9380663; doi:10.1007/s10389-022-01747-7)
Supplement: Supplementary file 2 — (DOC 96 kb) [file 10389_2022_1747_MOESM2_ESM.doc]

**
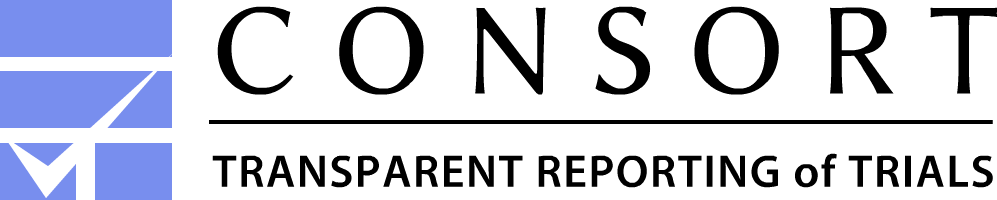
**

**CONSORT 2010 Flow Diagram**

**Randomization**

**Analysis**

**Follow-Up**

**Enrollment**

Assessed for eligibility (n = 345)

Excluded (n = 0)

- all participants met the inclusion criteria

**Analysed (n = 41)**

- Excluded from analysis (incomplete data throughout the longitudinal section due to not filling out the online questionnaire) (n = 132)

**Follow-up at Z1, n = 49**

- Lost to follow-up (didn’t fill out the online questionnaire) (n = 124)

**Follow-up at Z2, n = 49**

- Lost to follow-up n = 0

**Follow-up at T1, n = 41**

- Lost to follow-up, n = 8

**Follow-up at T2, n = 40**

- Lost to follow-up, n = 1

Allocated to intervention (n = 173)

**Follow-up at Z1, n = 47**

- Lost to follow-up (didn’t fill out the online questionnaire) (n = 125)

**Follow-up at Z2, n = 46**

- Lost to follow-up, n = 1

**Follow-up at T1, n = 31**

- Lost to follow-up, n = 15

**Follow-up at T2, n = 16**

- Lost to follow-up, n = 15

Allocated to control (n = 172)

**Analysed (n = 31)**

- Excluded from analysis (incomplete data throughout the longitudinal section due to not filling out the online questionnaire) (n = 144)

Randomized (n = 345)
